# Effects of Extracorporeal Photopheresis on Quality of Life and the Course of Diseases in Patients With Mycosis Fungoides and Graft-Versus-Host Disease: A Single-Center Analysis

**DOI:** 10.7759/cureus.38929

**Published:** 2023-05-12

**Authors:** Romanos Vogiatzis, William Krüger, Michael Jünger, Andreas Arnold

**Affiliations:** 1 Department of Dermatology, Greifswald University Hospital, Greifswald, DEU; 2 Department of Internal Medicine, Greifswald University Hospital, Greifswald, DEU

**Keywords:** immunosuppressants, quality of life, ecp, extracorporeal photopheresis, gvhd, mycosis fungoides

## Abstract

Introduction: The aim of the study was to systematically analyze the influence of extracorporeal photopheresis (ECP) on the quality of life (LQ) and the course of the disease in patients with Mycosis Fungoides (MF), as well as with Graft-versus-Host Disease (GvHD).

Methods: LQ was monitored retrospectively by using the dermatology life quality index (DLQI) and Skindex-29 test before ECP onset and after the last ECP. Disease parameters were assessed by objective criteria i.e. number of associated medical drugs taken, intervals between therapeutic cycles, gradual change of the disease, and eventual side-effects and complications of ECP therapy.

Results: Fifty-one patients were treated with ECP during 2008-19; 19 out of 51 died, and follow-up was not completed in 13 patients. Finally, treatment protocols of 671 ECP procedures were evaluated in 19 patients (10 MF; 9 GvHD). MF and GvHD subpopulations did not differ in the individual scores of LQ questions, either before the outset or after the last ECP. DLQI and Skindex-29 scores were ameliorated by the ECP therapy (p= 0.001 and p< 0.001, respectively) due to improvement of individual scores of feelings, daily/social activities (p< 0.05), and functionality (p≤ 0.05). The median interval between ECP cycles was extended from two to eight weeks (p= 0.001). Needs of GvHD patients for drugs being received for the underlying disease were reduced (p= 0.035). Two of the 10 MF patients worsened from stage IIA to IIIA. Severe or minor side effects leading to a therapy interruption were not recorded.

Conclusion: Patients with GvHD experienced a notable decrease in the administration of drugs for their underlying condition, and there were no instances of severe side effects that resulted in the discontinuation of treatment. ECP is safe and effective for the treatment of MF and GvHD.

## Introduction

Extracorporeal photopheresis (ECP) is a process of extracorporeal irradiation of blood components with ultraviolet-A (UVA) light, whereby the effectiveness is increased by the addition of a photosensitive substance. This therapy, which combines leukapheresis with photo-chemotherapy, was first applied in dermatology 30 years ago [1]. The ECP uses the immune system, modulating the therapeutic effect of photochemotherapy on the skin and expands this effect to the patient's tumor cells, which circulate in the blood. The exact mechanism of action of the ECP is not yet fully understood. Two different types of reactions have been detected: (1) Photo-activation of 8-methoxypsoralen (8-MOP) by UVA irradiation results in anti-proliferative effects (inhibition of mitosis, reduction of ribosomal activity) in the irradiated cells due to interference with the DNA. The latter results in the formation of crosslinks that inhibit the transcription and replication of DNA [2]; (2) Oxygen-dependent immediate reaction forms free radicals which destruct cell membranes. In addition, there is an inhibition of the transcription of interleukin 16 and 8+ as well as TNF-α, which leads to the anti-inflammatory and anti-proliferative effects of ECP therapy. Interleukin 1 and 8, control the migration of T-lymphocytes, while interleukin 1 and 6 represent accessory signals in the context of T-cell activation [1].

It has been suggested that ECP therapy, unlike other immunosuppressive regimens, does not cause global immunosuppression, but induces immune tolerance. Clinical and animal studies demonstrate that ECP therapy induces antigen-specific regulatory T-cells, including CD4+CD25+FoxP3+ T-cells and interleukin (IL)-10-producing Tr1 cells, that may arise secondarily to the induction of tolerogenic antigen-presenting cells by infusion of apoptotic cells [3,4]. The ECP was first developed and permitted for the therapy of cutaneous T cell- lymphomas (CTCL) [1], but in the meantime, ECP has established itself for the therapy of the Graft-versus-Host disease of skin (GvHD) [4]. Other indications are rejection reactions of heart and lung transplants, systemic sclerosis, and severe atopic dermatitis. The advantages of ECP are not only the improvement of the quality of life but also an extended lifetime of treated patients [5]. In contrast, high costs and a lot of work expenditure have to be mentioned.

Retrospective studies have been carried out in the last years and have evaluated the possible side effects [6] as well as the optimal initiation scheme of ECP therapy [7]. Overall, the ECP is a safe therapy, which rarely leads to serious side effects i.e. hypotension, vasovagal syncope, and anemia [6,8]. Due to the use of anticoagulation with heparin during the therapy, heparin-associated side effects, like heparin-induced thrombocytopenia type II (HIT 2), have to be considered [9].

The aim of this retrospective study was to systematically investigate the influence of ECP on the quality of life and on the course of the disease in patients with MF as well as with GvHD. For the purpose of the study, the state of quality of life was monitored by evaluating two questionnaires, and the course of the disease was assessed by using digital patient data records.

## Materials and methods

The study is a single-center retrospective analysis of patients with clinical symptoms of confirmed MF or GvHD, who were treated by ECP therapy from January 2008 until December 2019 in the dermatological clinic of the Greifswald University Hospital. After obtaining the Institutional Ethics Committee approval (approval number BB 100/19) on 30.07.19 and patients' consent, we studied patients aged between 18 and 85 years, with sufficient German language skills. Pregnant women and patients with incomplete elicitation of data were excluded from the study.

Design of the study

The main target was the comparative retrospective evaluation of the severity of symptoms of MF and GvHD, before the initiation of ECP therapy and after the last cycle of ECP therapy, by using the dermatology life quality index (DLQI) [10], and the Skindex-29 test for the assessment of patients’ everyday life [11]. The secondary target was the assessment of the disease’s pattern, which has been measured by objective criteria i.e. the number of associated medical drugs taken, the therapeutic intervals (intervals between the cycles), the clinical manifestation of the skin disease, the gradual change of the disease, and eventually the side-effects and complications of ECP therapy. Additionally, in GvHD patients were assessed the values of thrombocytes and liver function tests.

Procedure of ECP

The technical procedure for the extracorporeal photopheresis (ECP) performed in this study is described in the German guidelines [12]. Typically, a whole blood sample was obtained preferentially by a peripheral or alternatively by a central vein. During the leukapheresis, the blood was centrifuged in order to separate the plasma’s erythrocytes from the leucocytes. The 8-MOP sterile solution (Uvadex, 20 mg/ml, Therakos Europe, Ascot, UK) was added to the leucocytes fraction, the so-called buffy coat and the formed mixture was radiated with UVA light. At the end of the photo-activation time, the treated blood components were reinfused to the patient. ECP treatments were always carried out on two consecutive days. Routine laboratory tests (blood count, coagulation studies, and biochemical exams of liver and kidney function) were performed before every cycle of the ECP. The UVAR XTS photopheresis system (Therakos, Inc. Johnson & Johnson, NJ) was used from 2008 to 2015 and was replaced by the CELLEX system (Therakos, Inc. Johnson & Johnson, NJ) in 2015. There are no known differences in the effectiveness of these two procedures. The data of the treatments (e.g. time intervals between cycles of ECP, irradiated volume, duration of therapy, time of photoactivation) have been archived for every ECP session according to the treatment protocol. 

Recording of quality of life

The quality of life of the patients has been recorded retrospectively, using questionnaires (Skindex-29 and DLQI). The Skindex-29 questionnaire has been developed in order to measure the effects of skin diseases on patients’ quality of life and the DLQI is an established questionnaire for the recording of the quality of life of patients with dermatological disease patterns. The first version of Skindex consisted of 61 items, of which eight scales were classified [13]. The improved and revised version of the questionnaire is shorter and shows good reliability and validation. It consists of 29 items, which are distributed on three scales; a scale “symptom” with seven items, a scale “emotion” with 10 items, as well as a scale “functioning ability” with 12 items. The patient chooses from a 5-subject graded scale (never/ rarely/ sometimes/ often/ always) the best statement for him/her [11,14]. The DLQI [10,13] is an established questionnaire to assess the quality of life of patients with dermatological disease patterns, which encompasses 10 items with 4 grades, starting from 0 (not at all) until 3 (very much). At first, the patients were interviewed about their symptoms before the introduction of the ECP therapy, and then they were questioned about their current situation, during the period 2019-2020, after the application of the last ECP cycle. In the current study, we used validated versions of both questionnaires in the German language [11,15].

Statistical analysis

Descriptive statistics are used. Median values with interquartile ranges of the non-parametric measures and mean values with standard deviations of the parametric measures are presented. Data of DLQI and Skindex-29 appear as scores. The average absolute values were recorded in Excel spreadsheets (Microsoft Corporation, USA, Redmond, WA). The evaluation of the questionnaires was carried out by the database software Microsoft Access (Microsoft Corporation, USA, Redmond, WA). Paired comparisons between nonparametric data were conducted using Wilcoxon signed rank test. Comparisons between two independent groups of categorical data were conducted using the Chi-square test. For each test, P< 0.05 was considered statistically significant. The statistical analysis was carried out through the IBM-SPSS Statistics 24 software (IBM Corp., Armonk, NY).

## Results

**Figure 1:**
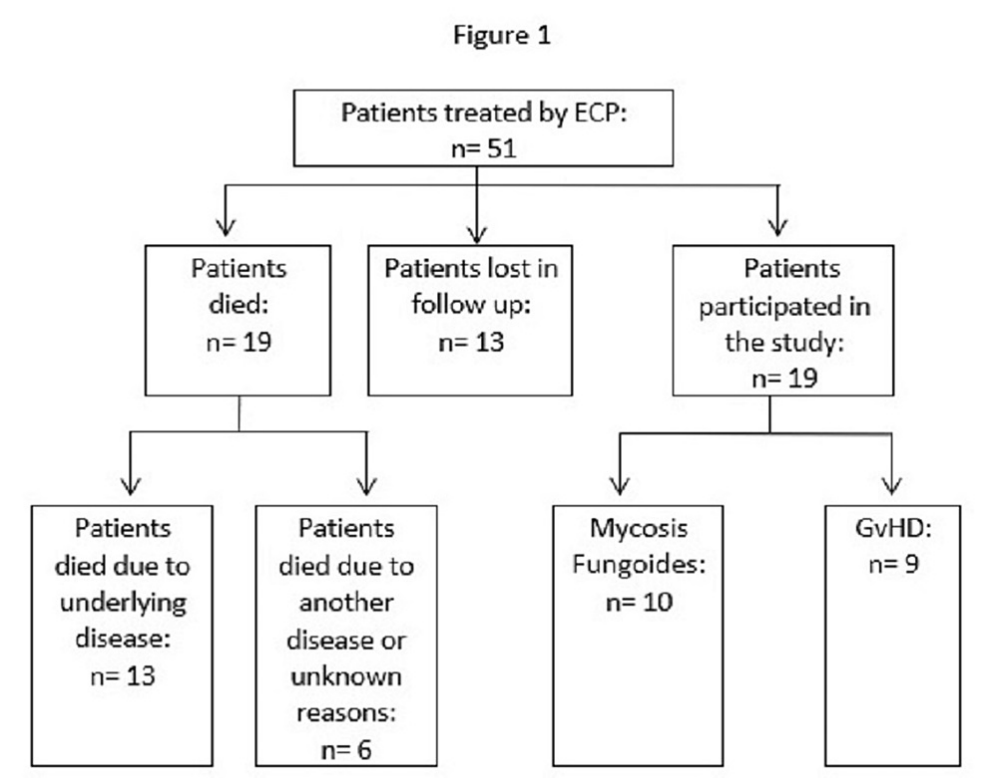
Study flow diagram illustrating patients included for analysis

Fifty-one patients have been treated with ECP from January 2008 until December 2019 in the clinic of Dermatology, Venereology, and Allergology of the University Hospital of Greifswald most of them over years and with multiple treatment cycles. Twenty-two patients were diagnosed with MF, 21 with GvHD, and eight with other diseases (chronic lung rejection after lung transplantation, Sezary-syndrome, systemic sclerosis). Nineteen of the 51 patients died and in 13 patients the follow-up was not completed. Figure 1 explains the study flow diagram of patients included for analysis (n= 19). Treatment protocols of 671 ECP procedures were evaluated.

Table 1 shows the demographic data of patients, parameters of ECP therapy (year of initiation, number of years that ECP was applied, total number of ECP therapeutic cycles) and therapeutic effects recorded (duration of intervals between ECP therapeutic cycles, number of medical drugs given).

**Table 1:** Demographic data and distribution of parameters of ECP therapy ECP: Extracorporeal photopheresis; MF: Mycosis fungoides; GvHD: Graft-versus-Host-Disease

Disease & Patient No.	Age (Yrs)	Gender (M/F)	Initiation of ECP (Year)	Total No. of ECP cycles	Years of ECP
MF 1	64	M	2011	31	9
MF 2	81	M	2018	17	2
MF 3	79	M	2008	100	12
MF 4	66	F	2015	41	5
MF 5	69	M	2017	25	3
MF 6	49	F	2017	31	3
MF 7	62	M	2008	56	12
MF 8	41	M	2015	56	5
MF 9	55	M	2011	67	9
MF 10	82	F	2011	15	7
GvHD 1	66	M	2008	75	12
GvHD 2	64	M	2016	37	4
GvHD 3	66	M	2017	28	3
GvHD 4	68	M	2018	10	2
GvHD 5	55	F	2017	18	3
GvHD 6	71	F	2008	38	12
GvHD 7	64	F	2019	5	1
GvHD 8	62	M	2014	19	3
GvHD 9	60	M	2014	3	1
Mean value ± SD	64.4 ± 9.9			35.4 ± 24.9	
Upper quartile					9
Median					4
Lower quartile					3
Range	41 – 89			3 – 100	1 – 12

Table 2 shows the indirect therapeutic effects recorded (duration of intervals between ECP therapeutic cycles, number of associated medical drugs given). Initially, the patients had a median ECP therapy interval of two weeks and at the end of the study the median interval was significantly extended to eight weeks (Wilcoxon signed rank test: p=0.001, z=-3.43). In four patients with MF (40%) and three with GvHD (33%), a reduction in immunosuppressant drugs received for the underlying diseases was determined. However, this reduction was statistically significant only for the patients with GvHD (Chi-square test: p=0.035). Patients with MF did not need to increase their medication.

**Table 2:** Therapeutic effects of ECP therapy ECP: Extracorporeal photopheresis; MF: Mycosis fungoides; GvHD: Graft-versus-Host-Disease

Disease & Patient No		Intervals of therapeutic cycles (weeks)		No. of associated medical drugs			
		Outset of ECP	At the end	Outset of ECP	At the end		
MF 1		2	8	0	0		
MF 2		2	12	1	0		
MF 3		4	16	0	0		
MF 4		4	6	1	0		
MF 5		4	11	1	1		
MF 6		2	6	2	1		
MF 7		4	12	2	1		
MF 8		2	3	1	1		
MF 9		4	8	0	0		
MF 10		4	8	1	1		
				0.07, NSS**			
GvHD 1		5	15	2	2		
GvHD 2		2	8	3	3		
GvHD 3		2	12	3	1		
GvHD 4		2	12	0	0		
GvHD 5		2	12	2	2		
GvHD 6		8	3	0	0		
GvHD 7		3	4	1	0		
GvHD 8		2	16	1	0		
GvHD 9		2	2	0	0		
	0.001, z=-3.43 *			0.035**			
Upper quartile		4	12				
Median		2	8				
Lower quartile		2	6				
Range		2 – 8	2 – 16				

Table 3 illustrates that 16 of 19 (84.2%) patients have shown an improvement of the subjective symptoms of the diseases assessed by Skindex-29, and in three of 19 (15.8%) patients (MF10, GvHD1, GvHD8) the symptoms had been stabilized. Finally, none of the patients has noticed a deterioration of the subjective symptoms. Skindex-29 scores were ameliorated by the ECP therapy (Wilcoxon signed rank test: p<0.001, z=-3.52). 

**Table 3:** Distribution of Skindex-29 scores ECP: Extracorporeal photopheresis; MF: Mycosis fungoides; GvHD: Graft-versus-Host-Disease; Skindex-29: A validated measure of the effects of skin diseases on patients' quality of life

Disease & Patient No.	Skindex-29 score	
	Before ECP	At the end
MF 1	82	34
MF 2	76	66
MF 3	116	67
MF 4	57	51
MF 5	45	39
MF 6	88	69
MF 7	111	31
MF 8	76	68
MF 9	71	59
MF 10	38	38
GvHD 1	45	45
GvHD 2	109	87
GvHD 3	114	43
GvHD 4	142	132
GvHD 5	95	47
GvHD 6	96	89
GvHD 7	102	87
GvHD 8	36	36
GvHD 9	94	60
	P< 0.001, z= -3.52*	
Upper quartile	105.5	68.5
Median	88	51
Lower quartile	64	38.5
Range	36 – 142	31 – 132

Table 4 illustrates that 13 of 19 (68.4%) patients have shown an improvement in the subjective symptoms of the diseases assessed by DLQI, and in six of 19 (31.6%) patients (MF4, MF5, MF8, MF10, GvHD1, GvHD8) the symptoms had been stabilized. Finally, none of the patients has noticed a deterioration of the subjective symptoms. DLQI scores were ameliorated by the ECP therapy (Wilcoxon signed rank test: p=0.001, z=-3.18). 

**Table 4:** Distribution of DLQI scores ECP: Extracorporeal photopheresis; MF: Mycosis fungoides; GvHD: Graft-versus-Host-Disease; DLQI: Dermatology Life Quality Index

Disease & Patient No.	DLQI score	
	Before ECP	At the end
MF 1	11	0
MF 2	11	3
MF 3	23	1
MF 4	2	2
MF 5	2	2
MF 6	9	3
MF 7	16	0
MF 8	10	10
MF 9	13	9
MF 10	0	0
GvHD 1	3	3
GvHD 2	23	4
GvHD 3	25	0
GvHD 4	22	19
GvHD 5	11	1
GvHD 6	8	7
GvHD 7	21	13
GvHD 8	1	1
GvHD 9	8	7
	P= 0.001, z= -3.18*	
Upper quartile	18.5	7
Median	11	3
Lower quartile	5.5	1
Range	1 – 25	0 – 19

Tables 5-6 reveal the distributions of P-values of Wilcoxon signed rank tests applied to compare the scores of the individual Skindex and DLQI questions between MF and GvHD patients, either before the outset of ECP, or after the last ECP. Out of the 80 questions in total (DLQI and Skindex pre-and post-ECP), only the answer to the 7th question of the Skindex after the last ECP seems to differ between MF and GvHD patients. Therefore, the two subpopulations (MF and GvHD patients) can be unified in order to evaluate the differences in the evaluation of the quality of life before and after the ECP treatment.

**Table 5:** Distribution of P-values of Wilcoxon signed rank tests applied to compare the scores of the individual Skindex questions between MF patients and GvHD patients, either before the outset of ECP (Pre), or after the last ECP (Post) ECP: Extracorporeal photopheresis; MF: Mycosis fungoides; GvHD: Graft-versus-Host-Disease; Skindex: Test to measure the effects of skin diseases on patients' quality of life

Skindex questions	MF vs. GvHD		MF vs. GvHD
	Pre		Post
1	0.50		0.18
2	0.17		0.50
3	0.56		0.32
4	0.83		0.85
5	0.16		0.34
6	0.14		0.15
7	0.06		0.01
8	0.62		0.25
9	0.59		0.38
10	0.45		0.96
11	0.77		0.36
12	0.48		0.26
13	0.78		0.22
14	0.37		0.58
15	0.78		0.80
16	0.56		0.74
17	0.83		0.29
18	0.55		0.96
19	0.81		0.42
20	0.53		0.22
21	0.60		0.12
22	0.54		0.23
23	0.19		0.41
24	0.84		0.77
25	0.78		0.59
26	0.15		0.39
27	0.74		0.56
28	0.70		0.48
29	0.61		0.49
30	0.86		0.26

**Table 6:** Distribution of P-values of Wilcoxon signed rank tests applied to compare the scores of the individual DLQI questions between MF patients and GvHD patients, either before the outset of ECP (Pre), or after the last ECP (Post) ECP: Extracorporeal photopheresis; MF: Mycosis fungoides; GvHD: Graft-versus-Host-Disease; DLQI: Dermatology Life Quality Index

DLQI questions	MF vs. GvHD		MF vs. GvHD
	Pre		Post
1	0.99		0.81
2	0.49		0.54
3	0.48		0.58
4	0.65		0.31
5	0.45		0.39
6	0.33		0.39
7	0.20		0.39
8	0.42		0.39
9	0.37		0.34
10	0.30		0.07

Tables 7-8 show the distribution of P-values of Wilcoxon signed rank tests applied to compare the scores of the individual DLQI and Skindex questions of the total number of studied patients (n= 19), between the outset of ECP and after the last ECP. 

**Table 7:** Distribution of P-values of Wilcoxon signed rank tests applied to compare the scores of the individual Skindex questions of the total number of studied patients (n=19), between two different times: before the outset of ECP (Pre) and after the last ECP (Post)

Skindex questions	Pre vs. Post
1	0.17
2	0.05
3	0.05
4	0.22
5	0.06
6	0.24
7	0.12
8	0.14
9	0.29
10	0.04
11	0.28
12	0.16
13	0.57
14	0.10
15	0.11
16	0.73
17	0.69
18	0.11
19	0.03
20	0.08
21	0.11
22	0.56
23	0.56
24	0.32
25	0.26
26	0.15
27	0.10
28	0.02
29	0.03
30	0.24

**Table 8:** Distribution of P-values of Wilcoxon signed rank tests applied to compare the scores of the individual DLQI questions of the total number of studied patients (n=19), between two different times: before the outset of ECP (Pre) and after the last ECP (Post)

DLQI questions	Pre vs. Post
1	0.07
2	0.002
3	0.17
4	0.04
5	0.02
6	0.11
7	0.12
8	0.11
9	0.19
10	0.19

Table 9 shows the clinical stage of MF patients. Two patients with MF (20%) were burdened in terms of the clinical stage, since they were at stage IIB initially, and while they were being treated with ECP their condition deteriorated by a transition into stage IIIB. Complete data about the clinical stage of patients with GvHD were not recorded in our data pool. 

**Table 9:** Clinical stage of patients with MF MF: Mycosis fungoides

	Initial	At the end
MF 1	IB	IB
MF 2	IIA	IIA
MF 3	IIA	IIIA
MF 4	IIA	IIIA
MF 5	IIIB	IIIB
MF 6	IIIA	IIIA
MF 7	IIIA	IIIA
MF 8	IIB	IIB
MF 9	IIB	IIB
MF 10	IIIB	IIIB

Table 2 illustrates that 13 of 19 (68.4%) patients have shown an improvement in the subjective symptoms of the disease, and in six of 19 (31.6%) patients (MF4, MF5, MF8, MF10, GvHD1, GvHD8) the symptoms had been stabilized. Finally, none of the patients has noticed a deterioration of the subjective symptoms. DLQI and Skindex-29 scores were ameliorated by the ECP therapy (Wilcoxon signed rank test: p=0.001, z=-3.18 and p<0.001, z=-3.52, respectively). 

Table 1 shows that in four patients with MF (40%) and three with GvHD (33%), a reduction in immunosuppressant drugs received for the underlying diseases was determined. However, this reduction was statistically significant only for the patients with GvHD (Chi-square test: p=0.035). Patients with MF did not need to increase their medication. Initially, the patients had a median ECP therapy interval of two weeks, and at the end of the study the median interval was significantly extended to eight weeks (Wilcoxon signed rank test: p=0.001, z=-3.43, Table 1). The median total duration of ECP application was four years (Table 1).

Table 3 and Table 5 reveal the distributions of P-values of Wilcoxon signed rank tests applied to compare the scores of the individual DLQI and Skindex questions between MF patients and GvHD patients, either before the outset of ECP, or after the last ECP. Out of the 80 questions in total (DLQI and Skindex pre-and post-ECP), only the answer to the 7th question of the Skindex after the last ECP seems to differ between MF and GvHD patients. Therefore, the two subpopulations (MF and GvHD patients) can be unified in order to evaluate the differences in the evaluation of the quality of life before and after the ECP treatment.

Consequently, Table 4 and Table 6 show the distribution of P-values of Wilcoxon signed rank tests applied to compare the scores of the individual DLQI and Skindex questions of the total number of studied patients (n= 19), between the outset of ECP and after the last ECP.

Table 7 shows the clinical stage of MF patients. Two patients with MF (20%) were burdened in terms of the clinical stage, since they were at stage IIB initially, and while they were being treated with ECP their condition deteriorated by a transition into stage IIIB. Complete data about the clinical stage of patients with GvHD were not recorded in our data pool.

There were no statistically significant differences in mean values of alanine transaminase (two-tailed T-test: p= 0.60), aspartate transaminase (two-tailed T-test: p= 0.88), and thrombocytes (two-tailed T-test: p= 0.95) in GvHD patients before ECP onset and after last ECP. Similarly, there were no statistically significant differences in median values of g-GT (Wilcoxon signed rank test: p=0.44, z=-.771).

No minor or other severe side effects were recorded. From our cohort, three patients (16%) stopped the therapy for personal reasons, i.e. two patients (Table 1: GvHD 8 and GvHD 9) invoked the expenditure to reach our clinic and one patient (Table 1: MF 10) invoked the long distance between the clinic and his residence. All other patients (84%) adhered to the treatment plan, without any delay and/or interruption of the ECP treatment, from the day of admission to the therapeutic program to the day of the scheduled data entry.

## Discussion

Photopheresis is an appropriate term for the procedure which incorporates exposure to UVA radiation, since in the Greek language “photo” means light and “apheresis” means subtraction (of blood’s components) [16]. Similarly, leukapheresis means the subtraction of “leuka”, as “leuka” means white (blood cells).

MF and GvHD affect more often the men [5]. Similarly, the general incidence of cancer diseases is about 20% more often in men than women [17]. Additionally, men have to be treated more often with stem-cell transplantation and they suffer more frequently from GvHD than women [18]. In terms of gender distribution, our data correspond to other larger series of patients [5].

The results of our study have shown that the median Skindex-29 score was decreased by 42% (from 88 to 51), after the application of ECP. A corresponding improvement between 40-50% has also been recorded by other retrospective studies [19-25]. The observed improvement in the Skindex-29 score mainly results from the improvement of the symptoms (questions 3, 10, 19, 28) and less from the improvement of the patients' functionality (questions 2, 29).

Our study has shown an improvement in DLQI, since the median score of the total number of patients observed was significantly decreased (3 compared with 11, an improvement of 72%), after the application of ECP. The observed improvement in the DLQI score results from the improvement of the feelings (question 2) and the daily and social activities (questions 4 and 5, respectively). ECP treatment does not seem to improve professional activity and interpersonal relationships. There is a sparsity of data regarding studies investigating the impact of ECP on DLQI, specifically in patients with MF. In a single-center prospective study [26] of 16 patients undergoing fortnightly ECP for moderate or severe chronic GvHD, DLQI assessments were carried out at baseline and after six months. The baseline median DLQI score was 7. Thirteen out of 16 patients (81%) showed an improvement in DLQI score, which was significantly lower after six months of ECP treatment (3.5 compared with 7, an improvement of 50%). Additionally, Flowers et al. [27] prospectively assessed the effect of ECP for 12 to 24 weeks on health-related quality of life in patients with cutaneous manifestations of chronic GvHD. In this study, the DLQI was not applied, but a Targeted Symptoms Assessment (TSA) questionnaire was used, and there was proven a numerically lower, but significant difference between the median improvement in TSA scores in the study group (with ECP: improvement 19%) compared with the control group (without ECP: improvement 2.5%). We used both questionnaires (DLQI & Skindex) because they are well established in the scientific community, and there are no relevant studies accessible to use simultaneously both.

In our study we assessed the course of treatment by recording the changes in the medication directly associated with the underlying disease, the intervals between the therapeutic cycles, the clinical characteristics of the skin disease and the change in stage of the disease.

The use of ECP may allow a significant reduction or even discontinuation of corticosteroids and/or other immunosuppressants, thus leading to reduced long-term morbidity and mortality and improved overall survival [28]. Therefore, the reduction and/or discontinuation of drugs associated with the underlying disease is a direct indicator of the benefit for patients mediated by the ECP. Assuming that disease activity can be depicted from the number of disease-specific drugs taken, we examined the quantity of the corresponding drugs received over time and recorded their changes numerically. Since the majority of these drugs are given in fixed doses, we considered that the recording of possible dose modifications of these drugs would be of secondary importance. In the Greifswald University Hospital, the following medications for the systemic treatment of the underlying diseases were used between 2008 and 2019: for MF patients interferon and bexarotene, and for GvHD patients prednisolone, ciclosporin and, more rarely, mycophenolate mofetil. In case of clinical improvement or stabilization of the course of the disease, the drug(s) was (were) discontinued completely. No patient had to discontinue the concomitant therapy due to side effects. Considering the small number of patients in each subgroup of underlying diseases (MF and GvHD), it appears that patients with GvHD clearly benefit from the ECP through a reduced number of medications they receive, while in the subgroup of patients with MF, the findings are marginal (0.05< P< 0.1) and obviously require a larger number of patients to derive a robust conclusion from it.

The extension of the intervals between the therapeutic cycles of ECP is an indirect sign of the improvement of the symptoms. The treatment schedule of patients with MF generally consists of two consecutive days every four weeks, with the aim of achieving a median duration of intervals between the ECP cycles of six months [29]. The respective regimen for chronic GvHD comprises three treatments a week for the first month, then two consecutive treatments every two weeks, and after that, the therapeutic goal is to achieve a median duration of intervals between the ECP cycles of six to nine months [29]. In our study, common initiation instructions were followed, both in the MF and in the GvHD patients, since the treatment schedule was generally initiated by two consecutive days every two weeks and the cycle intervals were extended solely because of the improvement in the local findings or patients’ general condition. Initially, the patients had a median cycle interval of two weeks, and at the end of the study, it was extended to eight weeks. This can indirectly show us that the patients benefited from the ECP since they required treatment less often.

Two patients with MF (20%) worsened from stage IIA to IIIA, although ECP is an effective and well-tolerated first-line therapy for the management of the early stages of MF [30]. We suspect an individual reaction in the sense of a “non-response” to the ECP therapy as a possible reason for the progression of the disease stage in patients with MF.

There is a sparsity of data regarding studies investigating the impact of ECP on liver function and platelet count. Our findings show that ECP does not cause any deterioration.

Several papers have reported on the safety proﬁle of ECP in the treatment of MF and GvHD. ECP is noted to be an extremely safe therapy concept and severe adverse events, such as vasovagal syncope, hypotension, anemia or infections secondary to indwelling catheters are rare. More than 500,000 treatments have been performed worldwide between 1987 and 2017 and the incidence of reported major adverse events is 0.003% [5]. The most common side effects are described as sporadic and mild, such as fatigue, hypotonic and hypertonic episodes, fever, headache, dizziness, dyspnea, or dysgeusia (mostly metallic taste), their total incidence is less than 3% [6-8]. The patients of our study did not present either serious or minor complications, and adhered to the treatment plan, without any delay and/or interruption of the ECP program.

The major limitation of the study is the retrospective design, meaning that asking questions about the conditions before the beginning of ECP therapy is more imprecise, as if the patients had been asked before the therapy was initiated.

## Conclusions

By means of retrospective evaluation of the questionnaires (DLQI and Skindex-29), we determined a subjective improvement in the quality of life of the examined patients, as the DLQI and Skindex-29 scores improved under the treatment of the ECP therapy. Initially, the patients had a median interval between the therapeutic cycles of ECP therapy of two weeks, and at the end of the study, the median interval was significantly extended to eight weeks. A significant reduction in the relevant drugs received for the underlying disease was confirmed for the patients with GvHD. Severe side effects leading to a therapy interruption were not recorded. Our findings suggest that ECP is safe and effective in the treatment of MF and GvHD.
